# IMU-Based Effects Assessment of the Use of Foot Orthoses in the Stance Phase during Running and Asymmetry between Extremities

**DOI:** 10.3390/s21093277

**Published:** 2021-05-10

**Authors:** Juan Luis Florenciano Restoy, Jordi Solé-Casals, Xantal Borràs-Boix

**Affiliations:** 1 Data and Signal Processing Research Group, University of Vic–Central University of Catalonia, 08500 Vic, Spain; juanluis.florenciano@uvic.cat; 2 Sport Performance Analysis Research Group, University of Vic–Central University of Catalonia, 08500 Vic, Spain; xantal.borras@uvic.cat

**Keywords:** running, kinematics, inertial measurement unit (IMU), foot orthoses, asymmetry

## Abstract

The objectives of this study were to determine the amplitude of movement differences and asymmetries between feet during the stance phase and to evaluate the effects of foot orthoses (FOs) on foot kinematics in the stance phase during running. In total, 40 males were recruited (age: 43.0 ± 13.8 years, weight: 72.0 ± 5.5 kg, height: 175.5 ± 7.0 cm). Participants ran on a running treadmill at 2.5 m/s using their own footwear, with and without the FOs. Two inertial sensors fixed on the instep of each of the participant’s footwear were used. Amplitude of movement along each axis, contact time and number of steps were considered in the analysis. The results indicate that the movement in the sagittal plane is symmetric, but that it is not in the frontal and transverse planes. The right foot displayed more degrees of movement amplitude than the left foot although these differences are only significant in the abduction case. When FOs are used, a decrease in amplitude of movement in the three axes is observed, except for the dorsi-plantar flexion in the left foot and both feet combined. The contact time and the total step time show a significant increase when FOs are used, but the number of steps is not altered, suggesting that FOs do not interfere in running technique. The reduction in the amplitude of movement would indicate that FOs could be used as a preventive tool. The FOs do not influence the asymmetry of the amplitude of movement observed between feet, and this risk factor is maintained. IMU devices are useful tools to detect risk factors related to running injuries. With its use, even more personalized FOs could be manufactured.

## 1. Introduction

Interest in the analysis of the foot strike pattern (FSP) has increased due to its association with a reduced risk of injury [1,2,3]. Furthermore, running gait pattern (GP) analyses can be used for injury prevention and treatment, as well as in performance enhancement [4]. GP and FSP have been analyzed using a variety of methods, such as two-dimensional (2D) video analysis, three-dimensional (3D) video analysis, center of pressure and force plate [5,6,7,8]. The 3D motion analysis system with cameras is considered to be the “gold standard”, but this system is expensive and complicated to use, requiring a set of reflective markers located on the appropriate reference points to be able to calculate the desired parameters [3,5,9].

Nowadays, there is a growing interest in evaluating GP and FSP in environments outside of the laboratory, and inertial measurement units (IMUs) are an interesting option to do so due to their reduced size and wireless properties [10]. An IMU sensor is the wearable device that will be used in our experiments to calculate the dorsi–plantar flexion (D–PF), the abduction–adduction (ABD–ADD) and the eversion–inversion (EV–INV) movements of the foot, using a combination of data from a triaxial accelerometer, a gyroscope and a triaxial magnetometer. The IMU sensor devices have attractive features such as low cost, low power consumption and overall simplicity in their use, and allow for the continuous monitoring of the subject’s daily activities, such as walking or running, either on a treadmill, or in real environments [10,11], but they also suffer from some disadvantages such as interference or measurement errors that must be taken into account. IMU devices have been used to determine gait kinematic changes when running [3,11,12,13,14,15,16,17,18,19], and several research studies have evaluated the reliability of IMU devices. For example, Boutaayamou et al. [12] validated the use of accelerometers, fixed on each shoe at the level of the heel and the proximal part of the big toe, against a 3D optical analysis system. Giandolini et al. [13] compared the use of accelerometers, placed on the external faces of the shoes at the heel and metatarsal levels, with the use of 2D video analysis. They compared the time between heel and metatarsal accelerations, as well as the foot strike angle, and it was determined that the method is reliable for a wide range of velocities. Sinclair et al. [17] concluded that accelerometers placed on the distal tibia can be used to precisely detect events during walking. Finally, Shiang et al. [19] used two IMUs (one accelerometer and one gyroscope) on the upper part of the shoe to determine the foot strike angle during running and found a significant correlation between the strike angle and the sagittal plane angles, which were acquired from a 3D system.

Running is the natural evolution of gait when increasing the velocity of movement. A stride can be divided into a stance phase, which occurs between foot strike and toe off, and a swing phase, which includes the double float, and the stance phase of the contralateral limb [4]. Running is one of the most popular physical activities, but it is also an activity with relatively high injury rate, with an estimation of one injury per year in 50% of all runners [20]. It is well known that injury occurrence is multifactorial [21], and, in running, there are several predisposing risk factors including: increased vertical ground reaction force relative to walking (2.2 times body weight after heel contact in running compared to 1.1 times body weight during walking) [4], long running distances, history of previous injury, cavus feet, muscle weakness, excessive supination during stance phase and bilateral asymmetry [20,21,22,23]. Previous references have highlighted that having a limb asymmetry greater than 15% is associated with an increased incidence of injury in both athlete and non-athlete populations [24,25].

Athletic foot orthoses (FOs) are shoe inserts that replace the removable stock insole. They have proven to be effective as a treatment for sport-related injuries and health diseases [20,26,27,28,29]. For example, Mündermann et al. [27] ascertained that the use of FOs reduced the maximum foot eversion and the ankle inversion moment, modifying the vertical loading rate and the maximum knee external rotation moment, and Lack et al. [28], through the placing of anti-pronation FOs, observed a significant decrease in hip adduction and in the internal rotation of the knee after the foot strike in subjects with severe pronation. Furthermore, Brognara et al. [29] observed that foot plantar stimulation using a 3D-printing insole generated more stable walking pattern in Parkinson’s disease patients.

It has also been observed that FOs could be a part of the armor in the prevention of running injuries [20,30,31]. For example, Franklyn-Miller et al. [31] evaluated foot pressures in four hundred aspiring military officers when running using pressure insoles. Out of the two groups, the subjects in the group who used personalized foot orthoses reduced overall injuries and stress fractures but not soft-tissue injuries. All of the studies referred analyzed parameters that are produced in the stance phase of the gait, so this is the phase chosen for analysis in the present study.

Healthy gait is assumed to be symmetrical, but asymmetries often exist [23,32]. Vagenas and Hoshizak [23] suggested that running shoes could significantly decrease the degree of rearfoot asymmetry, but no study has been found on the effect of FOs over the asymmetry of running.

Most articles focus on analyzing the effect of FOs from the perspective of injury [20,26,27,28,29], but this article does so from the perspective of non-injury and, therefore, prevention. The aim of this study is twofold: (1) to determine kinematic differences and asymmetries between feet during stance phase; (2) to evaluate the effects of FOs on foot kinematics. For the first objective, it is hypothesized that the kinematics of the foot will be symmetrical, and for the second objective, it is hypothesized that there will be no differences in foot motion when FOs are used.

As far as we know, this kind of study has never been conducted before with such a considerable number of subjects. Hence, the obtained results are highly useful and will allow us to have a deeper knowledge of the GP and the real effects of FOs in the stance phase of the stride.

## 2. Materials and Methods

This is a quantitative observational study following STROBE methodology [33].

The sample was composed by 40 male adult participants (age 43.0 ± 13.8 years; height 175.5 ± 7.0 cm; weight 72.0 ± 5.5 kg), regular amateur runners, that had been using FOs for at least one year. None of the subjects presented any alterations in their locomotor system.

All the subjects were informed of the conditions of the study and signed an informed consent prior to their participation. All the tests were non-invasive and followed the principles of the Declaration of Helsinki [34]. The study was approved by the University of Vic—Central University of Catalonia’s Ethical Committee (UVic-UCC, 09/2016).

### 2.1. Data Acquisition Procedure

Two IMUs, equipped with a triaxial accelerometer, a gyroscope and magnetometer (MotionPod, sampling rate of 30 Hz, size 33 × 22 × 15 mm, a weight of 14 g, Grenoble, France), along with a wireless interface (2.4 GHz, transmission range of up to 30 m, ≈8 h of usage, Grenoble, France) were used to collect the data. The software used was Logiciel Medical (RM Ingenierie, Rodez, France) [35,36]. This is a commonly used IMU in podiatry and physical therapy, with factory default settings. It has been shown to have the potential to assess movement and coordination variability between and within individuals from joint angle measures in swimming and limb orientation time-series data in climbing [37].

The analysis procedure started with the placing of the sensor on the instep of the subject’s sports footwear, using Velcro, which was then secured with adhesive tape to reduce vibrations in the device. Each participant brought their own footwear that they used regularly for running. It has been observed that changes in the hardness of the sole of the shoe can affect the kinematics of the inferior extremities [38,39]. The same footwear had to be used in the two evaluated conditions.

Each participant carried out a warm-up run on the running treadmill (BH Fitness G6414V SPORT, Álava, Spain) by running for three minutes at 2.5 m/s (9 km/h) without any inclination (measured with a bubble level) to familiarize themselves with the machine speed and the environment. It has been previously observed that running on a treadmill is representative of overground running [40,41]. Once the warm-up period had been completed, the participant rested for two minutes, and the rest of the experimental procedure was then explained.

**Figure 1:**
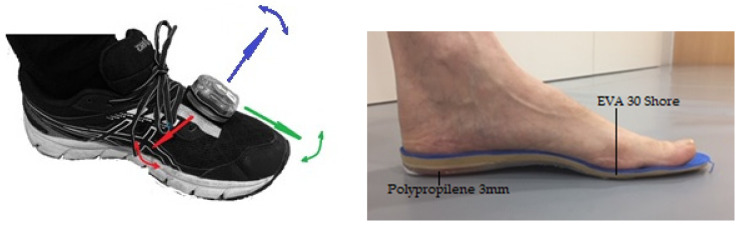
(**Left**): position of the sensor attached to the instep of the running shoe. The triple orthogonal system represented by the arrows indicate the dorsi–planar flexion (red), abduction–adduction (blue) and eversion–inversion (green) movements. (**Right**): type of FOs used with the polypro-pylene and the EVA layers.

According to the manufacturer’s protocol and instructions, the participant first remained still and upright for 3 s while the sensor was being calibrated. Thus, in a triple orthogonal relative reference system, the vertical axis collects the ABD–ADD movements of the foot, the longitudinal axis collects the EV–INV movements, and the D–PF movements are collected in the medio-lateral axis (Figure 1).

Considering that the transition between walking and running occurs at approximately 2.2 m/s [4], a low running speed of 2.5 m/s was chosen to meet the IMU sampling rate requirements. Consequently, two runs at 2.5 m/s were carried out, the first one with the subjects using their regular sports footwear and the second one wearing their regular sports footwear and FOs. As the effects of the FOs are mechanical and the acquisition time of the IMUs was 20 s, it was decided not to randomize the tests as it is considered that there is no familiarization effect in the run. Furthermore, we must take into account that the runners included, although recreational, have experience and accumulated mileage, and are already familiar with the FOs.

Following the IMU manufacturer’s recommendations, data acquisition was performed for 20 s after the treadmill speed stabilized. This time is guaranteed by MotionPod to ensure data reliability and avoid measurement errors caused by data integration over time [35]. The initial steps when transitioning between stationary and running phases, as well as the deceleration steps at the end, were discarded.

The manufacturing system of the FOs was that of thermoforming, with adaptation through a vacuum chamber. In the structure of the foot support, the thermoplastic material Polypropylene (3 mm) was used, and the cushioning material placed on the Polypropylene was Ethylene-Vinyl Acetate (EVA) of Shore 30 hardness (Figure 1).

### 2.2. Data Processing

The data was processed using Matlab (Mathworks, Natick, MA, USA). Taking the movement in the sagittal plane (D–PF) as a reference, the stance phase was determined for each step, which lies between the points of maximum and minimum plantar flexion. Thus, the starting and ending points of the stance phase of each step in the frontal and transverse planes are also obtained.

**Figure 2:**
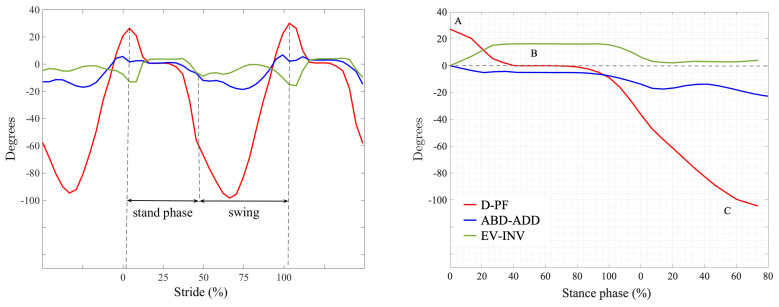
(**Left**): angular displacement of the foot as a function of the percentage of the running cycle. (**Right**): stance phase segmentation graph where A indicates the start of contact, B corresponds to the mid-stance (stabilization), and C indicates the end of the stance. Legend: D–PF indicates dorsi–plantar flexion, ABD–ADD indicates abduction–abduction, EV–INV indicates eversion–inversion.

Subsequently, a mathematical adjustment was carried out to ensure that in the D–PF, the plateau zone (mid-stance) corresponded with the 0° of the movement, and the possible deviations of the sensor in the movements of EV–INV and ABD–ADD were also corrected, adjusting the start at 0°. The swing phase was discarded in this study (Figure 2).

### 2.3. Data Analysis

The mean amplitude of maximum movement for each one of the axes and conditions was determined for the data analysis through the following functions:

(1) $$\bar{f}\left(t\right)=\frac{\sum_{i=1}^{N}f_{i}\left(t\right)}{N},\;\bar{a}\left(t\right)=\frac{\sum_{i=1}^{N}a_{i}\left(t\right)}{N},\;\bar{p}\left(t\right)=\frac{\sum_{i=1}^{N}p_{i}\left(t\right)}{N}$$

where $f_{i}\left(t\right)$, $a_{i}\left(t\right)$ and $p_{i}\left(t\right)$ are the functions representing the time evolution of the movement for D–PF, ABD–ADD and EV–INV movements, respectively; N refers to the number of steps taken by each subject during the 20 s of data collection.

Moreover, in the analysis between extremities, the asymmetry percentage was analyzed using Equation (2) [42]. This equation is considered to be accurate for the calculations of asymmetries in unilateral tests [43].

(2) $$Asymmetry=-100\;\frac{minimum\;value}{maximum\;value}+100$$

Matlab was used for the statistical analysis. Data normality was analyzed through the Kolmogorof–Smirnov test was assessed before doing the main test. The differences between the conditions were evaluated using a paired sample mean test (T-Student), confidence interval and the level of significant alpha were set at 95% and 0.05, respectively. To allow for a better interpretation of the data, the effect size (d-Cohen) was carried out with the following criteria: d≤0.2 negligible, 0.2≤d≤0.5 small effect, 0.5≤d≤0.8 medium effect, 0.8≤d large effect [44,45,46].

## 3. Results

### 3.1. Comparison between Feet

When comparing the kinematics between the feet (Table 1), it can be observed that the left and right feet present, on average, the same degrees of amplitude of movement in the sagittal plane (D–PF); in tenths of degrees, the right foot displays a smaller amplitude, although this difference is not statistically significant (*p* = 0.76), and the size of the effect is practically negligible (d = 0.05). In the frontal plane (EV–INV) and the transverse plane (ABD–ADD), the right foot exhibits slightly larger amplitude of movement values. These differences are not statistically significant for EV–INV (*p* = 0.09), but they are significant for ABD–ADD (*p* = 0.03). The size of the effect is small for both movements (d-EV–INV = 0.28; d-ABD–ADD = 0.37).

### 3.2. Comparison between Footwear with and without FOs

The data relating to the amplitude of movement, the time and the number of steps are presented in Table 2. The values are separated between the right foot, left foot and both feet.

It can be observed that the FOs decrease the values for the movement amplitudes, with the exception of the D–PF in the left foot and in both feet, which increase by a few tenths of a degree. This reduction in the amplitude of movement is statistically significant for the EV–INV in the left foot (p=0.02) and for the ABD–ADD in both feet (p=0.01). The size of the effect of the FOs is either negligible or small in both cases.

The differences in the time variables lie between 5 and 6 ms, significantly incrementing when the FOs are used. The number of steps is kept at a stable value for footwear with or without FOs.

The asymmetry between the extremities when FOs are used is of 5.3 ± 0.8% for D–PF, 24.8 ± 16.1% for EV–INV and 34.8 ± 18.2% for ABD–ADD. Here, no statistically significant differences are present in any case with the condition of the footwear without FOs (*p* = 0.07 for D–PF, *p* = 0.21 for EV–INV, *p* = 0.34 for ABD–ADD).

## 4. Discussion

### 4.1. Comparison between Feet

The null hypothesis established that there would be no differences between the feet in terms of the mean amplitude of movement.

The results (Table 1) indicate that the movement in the sagittal plane is symmetric, but not in the frontal and transverse planes. The right foot presents more degrees of amplitude of movement than the left foot in both IN–EVE and ABD–ADD, although these differences are statistically significant in the ABD–ADD case. These differences in foot kinematics are evidenced by the asymmetry percentages of IN–EVE and ABD–ADD of 21.7% and 31.9%, respectively.

Previous references have highlighted that presenting asymmetry between extremities that are bigger than 15% is associated with a higher incidence of injury, both in the athlete and non-athlete populations [24,25]. Then, the D–FP movement shows an asymmetry between extremities (6.2%) that is within the normal values, but the ABD–ADD and EV–INV show asymmetries that would represent a possible injury risk factor.

It is necessary to understand that the movements of the foot are not pure but combined. Thus, the pronation movement is carried out on three planes simultaneously: dorsal flexion, eversion and abduction. Similarly, the supination movement is carried out on the plantar flexion, inversion and adduction planes [47]. It has been observed that the eversion movement of the foot is present from the start of the stance phase until the beginning of the mid-stance, whereas the abduction movement is present from the mid-stance phase until the beginning of the swing phase. The start of the plantar flexion curve coincides with the start of the eversion and abduction curves. However, at the start of the contact, the plantar flexion and eversion curves present a larger slope than the abduction curve does, indicating that the movement is executed more quickly and that, therefore, the degree of eversion is directly proportional to the degrees of inversion before the start of the contact. Consequently, a bigger eversion is equivalent to a bigger abduction.

We have not found a study that compares the mechanics between extremities through the evaluation of the angular displacement. Nonetheless, other studies have also found differences between extremities using other types of mechanical variables. For example, Polk et al. [32] obtained gait asymmetries through the analysis of ground reaction force (GRF). They observed that vertical GRFs were very symmetrical, whereas there were significant asymmetries in the maximum mediolateral forces and impulses towards the dominant right limbs. In our study, the differences in lateromedial movements are also greater in the right foot, but we did not evaluate the laterality nor the lateral dominance of the subjects, which are two different concepts Carpes et al. [48] As a result, it cannot be known whether the observed differences are due to the predominance of the hand or to the dominant leg.

Cowley [49] analyzed the change in height of the medial foot arch after a 21 km run in 30 runners (12 women and 18 men), taking the navicular bone as a reference, and found a significant decrease in the foot arch in both feet (4.2 mm in the left foot, 5.0 mm in the right foot). Therefore, the study showed a change in posture of the foot, with a decrease in the medial arch, which was, again, more pronounced in the right foot, but did not give reasons for these changes. Stodółka et al. [50] examined the level of bilateral symmetry between the trajectory of the center of pressure (CoP) of the right and left feet in the latero-medial and antero-posterior directions. On the one hand, it was observed that 88% of the participants displayed symmetry of the left and right foot for the magnitude and direction of the antero-posterior trajectory of the CoP, but on the other hand, asymmetry was observed in 67% of the participants for the latero-medial trajectory; CoP displacement was noted along the lateral limit of one foot and along the medial limit of the other. Similarly, Montañola [51] discovered, in a study on 663 subjects, that the displacement, range and velocity of the CoP in the antero-posterior axis were bigger than in the latero-medial axis, and most of the subjects also showed a higher pressure on the right foot. On the other hand, De Carvalho et al. [52] obtained larger pronation values in the left foot than in the right foot in a male population using the foot posture index (FPI-6).

Rai et al. [53] registered footprints in 66 subjects, with and without a pathology, using an electronic pedobarograph. The results showed an asymmetric distribution of the plantar pressure in the right and left feet of the subjects without a pathology (17% had the same pressure on both feet, 7% had higher pressure on the left foot and 76% had higher pressure on the right foot).

In the rehabilitation of injuries, it is common to use the values of the contralateral leg as reference values. Nevertheless, data shown in the present study indicate that asymmetries in the kinematics of movement in non-injured people exist, supporting the results presented by Vagenas and Hoshiza [23] and Polk et al. [32]. Radzak et al. [54] also observed asymmetry in the angular values of the ankle, knee and hip in healthy subjects, as well as Gao et al. [55], who found asymmetry in the plantar pressure of the dominant extremity (the right extremity in all the subjects).

In summary, the asymmetry in the kinematic variables between the extremities during running can be seen in healthy subjects, which means that quantifying the possible differences and asymmetries could have implications in preventing injuries during walking, running and in the design choice of footwear.

It has been suggested that sidewalk running has greater kinematic variability than treadmill running [10], so it would be interesting to see, in further studies, whether these results hold outdoors in less cyclical and more variable conditions or whether they are even amplified. The technology used in this study could also be used to assess this, although a higher frequency of capture would be required.

### 4.2. Comparison between Footwear with and without FOs

The null hypothesis established that there would not be differences in the movement of the foot in favor of the FOs.

The results indicate (Table 2) that FOs decrease the amplitude of movement in the three axes, except for the D–PF in the left foot and both feet combined. For the D–PF movement, the differences are neither statistically significant in any of the two feet nor are they statistically significant when analyzing both feet combined. These results should be considered when talking about runners with functional hallux limitus as the FOs would further reduce the dorsiflexion of the first metatarsal. The less this movement, the more the foot is forced to take off in adduction [56].

The data obtained suggest that the FOs reduce the amplitude of movement of the EV–INV and ABD–ADD movements in both feet, although there is no significant difference agreement. If the number of subjects were to be increased, higher differences in these amplitudes of movement could probably be found. It is suggested that tendency is talked about instead of definite trends. Excessive supination during the stance phase has been observed as a risk factor for running injuries [20]. Therefore, a reduction in the amplitude of movement would reduce supination indicating that FOs could be used as a preventive tool. This is in accordance with Franklyn-Miller et al. [31] who found that the use of the personalized FOs reduced the frequency of injuries in a group of military officials undergoing training.

These results are in line with those observed in other studies [27,28,29,57,58]. For instance, Mündermann et al. [27] observed that the use of the FOs reduced the maximum foot eversion, while Lack et al. [28] observed a reduction in the hip adduction and in the internal rotation of the knee after foot strike in patients with severe pronation. Nawoczenski et al. [57] found that the use of FOs reduced the internal rotation of the tibia in the transverse plane.

While the present study did not evaluate the internal rotation of the tibia, we know that the tarsal joints (subtalar and transverse tarsal) connect with the tibia through the subtalar axis [58], which implies that the eversion of the foot (mid-stance phase) leads to internal rotation of the tibia. If we consider that in the mid-stance phase the action of the internal retro-malleolar muscles (especially the tibialis posterior) and the external retro-malleolar muscles (especially the peroneus longus) is eccentric, and that these muscles are responsible for the stabilization of the ankle in eversion and abduction, then the observed reduction could suggest a decrease in the activity and an increase in the efficiency of these muscles. Consequently, we suggest that by controlling this eversion mechanism, the internal rotation of the tibia could also be reduced. Moreover, the abduction movement is mainly produced in the impulse phase; therefore, reducing abduction would facilitate the impulse phase and with it, concentric muscle contraction of the aforementioned retro-malleolar muscles.

It has also been found in the literature that the use of FOs modifies other variables which have not been analyzed in this study. For example, Açak [59] compared the efficiency of personalized vs. prefabricated FOs and concluded that the individually designed FOs had a beneficial role in the normalization of the forces acting on the foot and improved the physical performance parameters of people with flat feet.

In the present study, the type of footprint (neutral, pronation and supination) of the subjects was not addressed, potentially masking, for example, a larger difference for the subjects with flat feet as Açak suggests [59]. Future studies could explore this issue.

The contact time and the total stride time present a significant increase when FOs are used. The differences, however, are, on average, 6 ms and do not affect the step frequency since the number of steps of the subjects analyzed did not change significantly with or without the use of the orthoses. These data suggest that the step length, which is directly proportional to the running velocity and inversely proportional to the step frequency, was also not modified. This is interesting, as even though FOs can modify the intrinsic kinematics of the foot, they do not interfere with the running technique.

Regarding asymmetry, it has been found that the kinematic changes occurred in both feet and that FOs had no effect on minimizing this risk factor. This is something novel which has not been described so far in the scientific literature. It is true that by varying the mechanics of the foot, the mechanics of the ankle, knee and hip are also affected [27,28,29,30,57,59], but this opens a new line of research in the fields of podiatry and sport medicine, which could lead to reducing the asymmetries found by personalizing the FOs even more.

## 5. Conclusions and Future Work

Based on the results of this study, we can conclude that (1) asymmetry is observed between the feet in the frontal and transverse planes, showing that the right foot has more degrees of amplitude of movement than the left foot. (2) There is a tendency for the FOs to reduce the amplitude of movement in the frontal and transverse planes, but not in the sagittal plane. (3) The kinematic changes observed using the FOs did not interfere with the technique of the run. (4) FOs do not influence the asymmetry of the amplitude of movement observed between the extremities, as the kinematic changes are produced in both legs.

IMU sensors are a good alternative for the study of locomotor system movement and can provide valuable data in a simple way. Moreover, IMU devices are useful tools to detect risk factors related to running injuries. Their generalized use would allow the manufacture of even more personalized and individualized FOs.

We are already now working on the collection of new data to expand our current sample of data and to determine if the preliminary results found can be confirmed. These new data are being collected for all the subjects and are related to their laterality and lateral dominance to be able to go more in depth in the subsequent analysis of the data.

## Figures and Tables

**Table 1:** Means and standard deviations of the angular displacement of each one of the extremities for each axis of movement.

	Left	Right	Asymmetry	*p*-Value	d-Cohen
Dorsal–Plantar Flexion	96.6 ± 13.5	96.2 ± 14.7	6.2 ± 5.2	0.76	0.05
Eversion–Inversion	12.2 ± 3.8	13.36 ± 3.9	31.9 ± 18.2	0.09	0.28
Abduction–Adduction	21.3 ± 7.4	25.4 ± 9.8	21.7 ± 14.5	0.03 *	0.37

Values for the angular displacement for each left and right leg in degrees. Values of asymmetry as a percentage. * Indicates statistically significant differences between angular values in the right and left legs (*p* < 0.05).

**Table 2:** Angular displacement, time variables and number of steps in each of the extremities (individually and for both feet) for each axis of movement.

		Footwear	Orthoses (FOs)	Difference	*p*-Value	d-Cohen
**Left**	Dorsi–plantar flexion (°)	96.6 ± 13.5	97.2 ± 12.1	0.62 ↑	0.42	−0.13
	Eversion–Inversion (°)	12.2 ± 3.8	11.5 ± 3.8	−0.77 ↓	0.02 *	0.37
	Abduction–Adduction (°)	21.3 ± 7.4	19.5 ± 6.9	−1.76 ↓	0.06	0.30
	Contact time (ms)	454 ± 41	459 ± 39	5 ↑	0.01 *	−0.42
	Total time (ms)	730 ± 54	736 ± 53	6 ↑	0.01 *	−0.40
	Number of steps	14.9 ± 1.5	14.8 ± 1.4	0.1 =	0.44	0.12
**Right**	Dorsi–plantar flexion (°)	96.2 ± 14.7	96.1 ± 13.5	−0.06 ↓	0.94	0.01
	Eversion–Inversion (°)	13.4 ± 3.9	13.2 ± 3.9	−0.17 ↓	0.75	0.05
	Abduction–Adduction (°)	25.4 ± 9.8	23.4 ± 8.9	−2.01 ↓	0.05	0.32
	Contact time (ms)	455 ± 42	461 ± 40	6 ↑	0.01 *	−0.47
	Total time (ms)	730 ± 55	736 ± 53	6 ↑	0.01 *	−0.39
	Number of steps	15.2 ± 1,3	15.0 ± 1.3	0.2 =	0.04 *	0,34
**Both**	Dorsi–plantar flexion (°)	96.4 ± 14.2	96. 7 ± 12.9	0.28 ↑	0.60	−0.06
	Eversion–Inversion (°)	12.8 ± 3.9	12.3 ± 4.0	0.47 ↓	0.13	0.17
	Abduction–Adduction (°)	23.3 ± 9.0	21.5 ± 8.2	1.89 ↓	0.01 *	0.31
	Contact time (ms)	454 ± 42	460 ± 40	6 ↑	<0.01 *	−0.45
	Total time (ms)	730 ± 55	736 ± 53	6 ↑	<0.01 *	−0.40
	Number of steps	15.0 ± 1.4	14.9 + 1.3	0.1 =	0.05	0.22

Mean and standard deviation for the variables. * Indicates statistically significant differences (*p* < 0.05).

## Data Availability

The datasets generated and analyzed during the current study are not publicly available due to ethics and privacy requirements but are available from the corresponding author on reasonable request.
